# Neuroproteomics of the Synapse: Subcellular Quantification of Protein Networks and Signaling Dynamics

**DOI:** 10.1016/j.mcpro.2021.100087

**Published:** 2021-04-29

**Authors:** Charlotte A.G.H. van Gelder, Maarten Altelaar

**Affiliations:** 1 Biomolecular Mass Spectrometry and Proteomics, Bijvoet Center for Biomolecular Research and Utrecht Institute for Pharmaceutical Sciences, Utrecht University, Utrecht, The Netherlands; 2 Netherlands Proteomics Center, Utrecht, The Netherlands

**Keywords:** neuroproteomics, synapse, protein networks, proximity-labeling, bio-orthogonal amino acids, mass spectrometry, protein translation, AHA, azidohomoalanine, AMPA, α-amino-3-hydroxy-5-methyl-4-isoxazolepropionic acid, AP-MS, affinity purification–MS, APEX, ascorbate peroxidase, BioID, biotin identification, CSPs, cell-surface proteins, FACS, fluorescence-activated cell sorting, HRP, horseradish peroxidase, iPSC, induced pluripotent stem cell, LCM, laser capture microdissection, LTD, long-term depression, mGluRs, metabotropic glutamate receptors, NMDA, N-methyl-D-aspartate, PPIs, protein–protein interactions, PRM, parallel reaction monitoring, PSD, postsynaptic density, PTMs, post-translational modifications, SILAC, Stable Isotope Labeling by Amino acids in Cell culture, SNO, S-nitrosylation, SRM, selected reaction monitoring, XL-MS, crosslinking MS

## Abstract

One of the most fascinating features of the brain is its ability to adapt to its surroundings. Synaptic plasticity, the dynamic mechanism of functional and structural alterations in synaptic strength, is essential for brain functioning and underlies a variety of processes such as learning and memory. Although the molecular mechanisms underlying such rapid plasticity are not fully understood, a consensus exists on the important role of proteins. The study of these neuronal proteins using neuroproteomics has increased rapidly in the last decades, and advancements in MS-based proteomics have broadened our understanding of neuroplasticity exponentially. In this review, we discuss the trends in MS-based neuroproteomics for the study of synaptic protein–protein interactions and protein signaling dynamics, with a focus on sample types, different labeling and enrichment approaches, and data analysis and interpretation. We highlight studies from the last 5 years, with a focus on synapse structure, composition, functioning, or signaling and finally discuss some recent developments that could further advance the field of neuroproteomics.

## Highlights

• The study of neuronal proteins using proteomics has developed rapidly.
• Focus on synapse structure, composition, functioning, or signaling.
• Isolation of single-cell types and organoids for ideal sample material.
• Increase in the use of bio-orthogonal and proximity labeling strategies.

## Graphical Abstract

**Figure:**
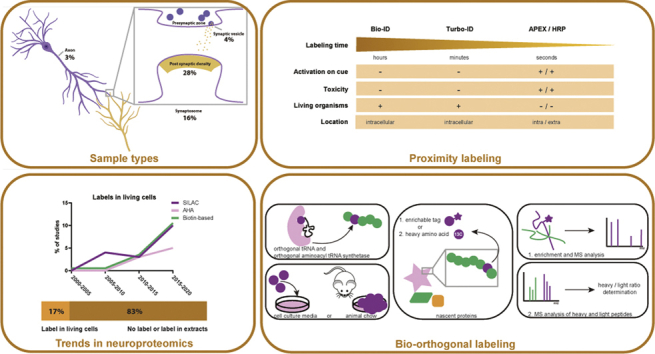

## In Brief

Advancements in MS-based proteomics have increased the study of synaptic proteins using neuroproteomics. The development of proximity, genetic labeling and bio-orthogonal amino acid labeling approaches now allow for the study of synaptic protein–protein interactions and protein signaling dynamics. In this review, we highlight studies from the last 5 years, with a focus on synapse structure, composition, functioning, or signaling and finally discuss some recent developments that could further advance the field of neuroproteomics.

Synaptic plasticity is defined as the dynamic process of functional and structural alterations in synaptic strength, where long-term potentiation implies the strengthening and long-term depression (LTD) the weakening of synaptic transmission. Dendritic spines, which harbor synapses, are highly abundant on forebrain dendrites, such that a single neuron can contain up to 10,000 synapses (2).

The huge dynamic alterations in spine composition demand the possibility of rapid protein synthesis, degradation, and trafficking (3, 4). Given the fact that the distance between the cell body and a spine can be enormous, these processes cannot be solely attributed to the transport from and to cytoplasmic organelle structures. In the last decade, a plethora of evidence has been gathered to support the existence of so-called satellite synaptodendritic organelles that would allow for fast and local turnover of proteins (reviewed in (5)).

There is overwhelming evidence on the occurrence of local protein synthesis in mature dendrites (reviewed in (6)), and recent efforts have supported the evidence in mature axons (reviewed in (7)). More than 75% of all excitatory and inhibitory presynaptic terminals were found to contain translational machinery, and distinct patterns of protein synthesis were observed in axonal terminals following three different types of synaptic plasticity (8).

The first synaptoneuroproteomics studies date from the early 2000s, when MS-based analysis of postsynaptic density (PSD) fractions was explored (9, 10, 11, 12, 13, 14, 15, 16, 17). These studies have revealed thousands of synaptic proteins and have been fundamental for the development of the neuroproteomics field. However, it is of fundamental importance to understand how synapses are organized not only physically, within synapses, but also spatially, between synapses. It is essential to invest in research focusing on brain areas other than classical synapse brain areas such as the hippocampus and cerebral cortex. Therefore, efforts have been made to compare PSD compositions of different brain regions and link their proteome signatures to both the anatomical region and embryonic origin (18). In the last decade, synaptic proteomics studies have increased significantly and have contributed to the understanding of brain function, development, and disease states, including a variety of mental disorders (reviewed in (19, 20, 21, 22)).

In this review, we highlight the trends in MS-based neuroproteomics based on studies from the last 5 years, with a focus on synapse structure, composition, functioning, and signaling. We describe several types of sample materials and their possible applications and benefits and drawbacks of their use. Next, we discuss isolation techniques to distinguish different cell types, such as astrocytes or specific types of neurons, and for the enrichment of subcellular fractions. We provide an overview of proteomics techniques to study protein–protein interactions (PPIs), protein synthesis and degradation, and protein signaling dynamics, with a focus on live cell proximity labeling approaches and the use of bio-orthogonal labeling approaches. Finally, we discuss recent developments in the field of protein analyses that could further advance the field of neuroproteomics.

## Neuroproteomics

### Sample Material

**Fig. 1:**
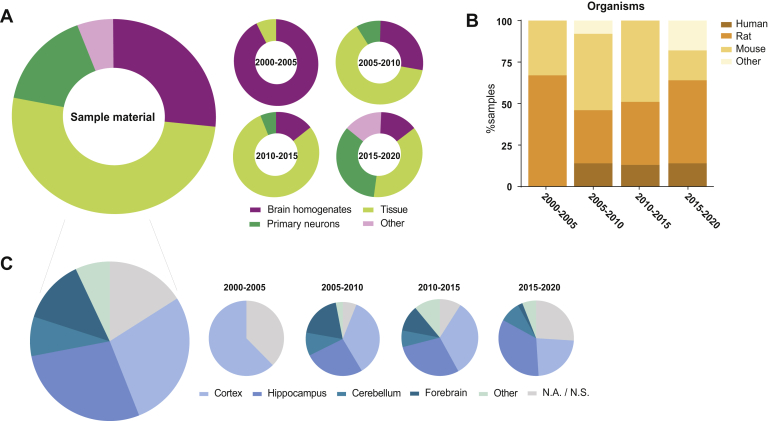
**Sample characteristics in neuroproteomics studies of the synapse.***A*, contribution of different sample materials. Although earlier studies were mostly performed on brain homogenates, increase in sensitivity of mass spectrometers resulted in a shift toward the use of tissue from specific brain regions and more recently toward more defined and homogeneous primary cultures. *B*, the most represented organisms in neuroproteomics studies are the rat and mouse. Although both brain homogenates and primary neurons are predominantly derived from rats, largely because of their larger brain size, genetically modified samples are often derived from mice. *C*, the distribution of the most studied brain regions has not significantly changed during the last decades.

In neuroproteomics experiments, the nature of the sample material inherently poses challenges for standard proteomics workflows. Limitations such as low sample amount caused by the use of terminally differentiated, nondividing cells and the heterogeneity in cell types have delayed the development of the neuroproteomics field. While earlier studies were mostly performed on brain homogenates, increased sensitivity of mass spectrometers resulted in a shift toward the use of tissue from specific brain regions and more recently toward more defined and homogeneous primary cultures (Fig. 1*A*). The most represented organisms in neuroproteomics studies are rats and mice (Fig. 1*B*). Studies of the early 2000s predominantly used rat brain homogenates, as these possess a larger brain mass. Over the years, a slight shift toward mouse models can be observed, which can be correlated to the increase in studies utilizing genetically modified samples. In the last 5 years, however, primary neuronal cultures derived from rats are increasingly used. Interestingly, the relative contribution of most studied brain regions, the hippocampus and the cortex, has not changed much in the last decades (Fig. 1*C*).

#### Postmortem and Surgical Tissue

Human brain samples originate from either neurosurgical biopsies (23, 24) or postmortem material (25, 26, 27) and are the preferred material source when studying the molecular fundaments in diseases with an unknown genetic origin. Postmortem material can be matched on sex, age, and several other characteristics but cannot always be controlled for genetic background, history of drug use, and comorbidity, increasing heterogeneity and thereby complicating data interpretation. Moreover, standardization of sample preservation remains challenging. With average response times of 4 to 7 h postmortem, protein modification and degradation are expected. Although this is less of a concern in surgically obtained material, where samples are dissected, classified into healthy or diseased tissue by an expert, and then snap-frozen to guarantee tissue stability, tissue heterogeneity remains an issue. Data interpretation could potentially be aided by the use of internal controls, where diseased and nondiseased tissue from the same patient is compared.

#### Neurocytometry

One technique with high potential to differentiate between different brain cell populations is fluorescence-activated cell sorting (FACS). Although widely applied to many different cell types, FACS in brain samples, the field of neurocytometry, has been of limited use (28). This was mainly caused by the need for genetic labeling of cell types (29), difficulties in generation of single-cell suspensions, and the lack of cell type–specific markers. For (synapto)proteomics purposes, the main limitation remains cell integrity during dissociation, where cell protrusions are not preserved and even cell bodies are commonly distorted to the point where many cytoplasmic proteins are lost and the main cell body consists of the nucleus (30). Moreover, common practice in human brain preservation includes formalin fixation or flash-freezing of tissue, both of which are incompatible with FACS. Recently published protocols, however, demonstrate functional neurocytometry for separation of different neuron types after fixation, with preservation of cytoplasmic proteins, and while maintaining RNA integrity (28). Other examples include fluorescence-activated synaptosome sorting, where mice are genetically modified with fluorescent glutamatergic synapses, enabling sorting of synaptosomes with a resealed presynaptic terminal and a PSD (31). These advances give promise to the use of neurocytometry in neuroproteomics in the future.

#### Laser Capture Microdissection

Another possibility for the isolation of specific brain cells is laser capture microdissection (LCM), in which a selection of a tissue of interest is excised using a UV or IF laser and captured in a collection tube. Advantages of LCM include the use of a wide variety of tissue preparations, its accurate separation of an extremely small number of cells, and even single-cell isolation. However, it is extremely time-consuming and relatively expensive (32). Its advantages over whole-tissue lysate analysis in proteomics studies are increased feature identification, increased peptide identification, and subsequent higher protein identifications and a decrease in missing values (33). Several studies have shown the use of LCM in combination with LC-MS/MS to study brain tissue abnormalities (34, 35).

#### Cultured Primary Neurons

One of the major advantages of culturing primary rat or mouse neuronal cells *in vitro* is that they synchronize before differentiation. This makes them very suitable for use in system-wide analyses, such as proteomics studies. Different stages of neuronal development, including axonal outgrowth, dendritogenesis, and ultimately the formation of synapses, can be followed in culture (36).

#### Induced Pluripotent Stem Cells

In the last decade, tremendous progress has been made in the use of induced pluripotent stem cell (iPSC)-derived neurons, allowing for cue-specific differentiation into dopaminergic (37, 38), serotonergic (39), and glutamatergic (40) neurons; several types of motor neurons were created (41), as well as cortical neurons (42), and various types of glial cells (43, 44). A major limitation in the use of these induced neurons was the representation of brain developmental state, where iPSC-derived neurons often reflect very early developmental stages (45). This problem was partially solved by protocols using small molecule cues for stepwise differentiation, allowing for the creation of phenotypically more developed neurons, characterized by processes such as synaptogenesis (46). In recent years, it has become possible to model synaptogenesis and synapse function in several diseases using iPSC-derived neurons (reviewed in (47)).

#### Species and Sex Specificity

Differences in synaptic protein profiles have been observed in a study comparing isolated synaptosomes from hippocampi of four different species. Two rodent proteomes, the rat and mouse, and two primate proteomes, the marmoset and human, were compared using sequential window acquisition of all theoretical fragment ion spectra MS (48). The most striking differences were observed between rodents and humans, and between marmosets and humans, whereas less variance in expression was observed between the two rodent species. Statistical analyses of a predefined set of plasticity-related proteins between the four species showed that especially proteins involved in endocytosis, ionotropic glutamate receptors, and auxiliary subunits were significantly expressed at lower levels in humans than the other three species. In contrast, components of the extracellular matrix were expressed at higher levels in humans. Recent findings by Sowers *et al.* indicate that also in synaptic disorders, differences can be observed between sexes. Using label-free quantification (LFQ) of hippocampal slices, they showed that in an *FGF14−/−* mouse model, the proteomic alterations were mainly sex specific and that the male proteome could be matched to readily available data from genome-wide association studies (49).

#### Cell-Surface Proteins

Over the past 2 decades, neuroproteomics has provided an incredible amount of data resulting in many new biological insights into the composition and functioning of synapses. However, the majority of studies have focused on intracellular proteins, or the intracellular interactome of transmembrane receptors, leaving cell-surface proteins (CSPs) vastly under-represented. CSPs pose several challenges for the classical proteomics workflows for a number of reasons, including their extensive post-translational modification (PTM) patterns and solubility in standard buffers, which mostly holds for transmembrane proteins. Secreted proteins add an extra challenge, as they require additional recovery steps (such as collection of cell culture media) and are often contaminated with proteins from different sources.

Interestingly, recent studies using single-cell sequencing strategies have discovered that different neuronal cell types present a unique set of CSP combinations. The landscape of proteins present on presynaptic and postsynaptic membranes, in the extracellular matrix, on glial membranes, as well as secreted proteins, results in the formation of different types of synaptic connections (as reviewed in (50, 51)). This notion is especially of interest in the study of connectivity between neurons and more generally the organization of the nervous system, as CSP patterning could hold key information on how and where two brain cells connect.

This was demonstrated in a recent article by Apóstolo *et al.* (52). In this study, mossy fiber synaptosomes were isolated *via* sucrose gradient centrifugation, making use of the mossy fiber characteristically large in size, where a large presynaptic bouton engulfs a series of postsynaptic densities or multiheaded dendritic spines. Analysis of synaptosomes from these mossy fiber microcircuits led to the discovery of more than 75 potential CSPs, most of which (almost 80%) were not previously reported to be localized or functional at the mossy fiber synapse, or any synapse, before. Moreover, the authors were also able to identify over 25 PPI pairs among the newly identified CSPs using a pairwise high-throughput interaction screen using an ELISA-based assay, yielding 38 interaction pairs, of which 10 were not reported before. To achieve this, the extracellular domain of all 73 potential CSPs were fused to alkaline phosphatase or the Fc region of IgG1 and all potential combinations of protein–protein pairs were tested. After a variety of validation experiments, IgSF8 was identified as a key regulator of the hippocampal CA3 microcircuit, emphasizing the importance of the inclusion of CSPs in neuroproteomics studies.

#### Contribution of Glia in Synapse Functioning

An important consideration in the data analysis and interpretation of neuroproteomics data is that the obtained information is derived from a mixture of different cell types. Most sample material is in fact comprised of a mix of neuronal cell types and several types of glia, being astrocytes, oligodendrocytes, and microglia. Glia were previously thought to serve merely as a supportive network for neuronal stability and functioning. However, exponential increase in the study of glial cells has highlighted pivotal roles of glia in nervous system development and in the maintenance of homeostasis by for instance the removal of dead neurons and pathogens (53). A plethora of experimental evidence now supports a pivotal role for glia in synapse formation and functioning. This was corroborated by the introduction of the ‘tripartite synapse’, where glia are now considered an integral part of the synapse, next to the presynaptic and postsynaptic parts of the neuron (54). The relevance of including glia in synapse proteomics studies was emphasized in a recent report where transcriptomic and proteomic data were combined to study the effect of the genetic duplication syndrome Dup15q in *Drosophila*, which often results in the development of pharmacoresistant epilepsy. In this study, the gene of interest was solely overexpressed in glia, and not in neurons. Interestingly, the combined analysis of transcriptomic and proteomics data showed downregulation of proteins that regulate synaptic transmission, including neurotransmitter secretion proteins (55). Another study showed that the neuronal cell adhesion molecule, expressed in astrocytes, interacts with its neuronal counterpart transcellularly. Moreover, loss of astrocytic neuronal cell adhesion molecules significantly decreased inhibitory synapses and their functioning (56).

These examples highlight the necessity of including glia in proteomics analysis of synapse formation and functioning. Current limitations in cell type–specific analyses have limited the number of ‘glioproteomics’ studies but will most likely gain tremendously by utilizing the combination of sophisticated genetic models and proximity-based proteomics profiling (discussed further on in this review).

In summary, the ideal sample for neuroproteomics studies consists of a single neuronal or glial cell type, as this removes uncertainty of the relative contribution of different cellular origins of proteins of interest that is common on brain lysates and tissues, and to a lesser extent, primary neurons. Genetic labeling of specific cell types and subsequent isolation is the most suitable approach to this aim, but lack of cell type–specific labels and low amounts of sample material limit their use. iPSC-derived neurons can be cultured on demand and have the advantage of a specific and human genetic background and can be labeled but lack the representation of real-life neuronal and glial networks. The fast-developing field of organoid technologies will likely lead toward the creation of the ideal proteomics sample material, as these contain all of the mentioned characteristics of the ideal neuroproteomics sample.

### Deciphering PPIs in Protein Networks

**Fig. 2:**
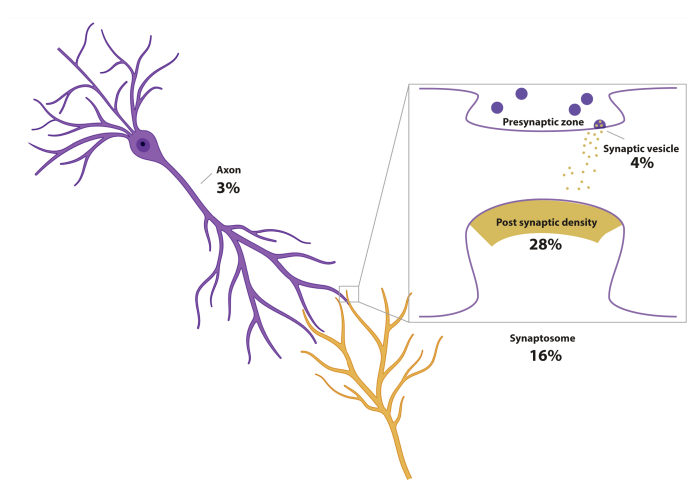
**The relative contribution of specific cellular compartments in studies utilizing subcellular fractionation techniques.** Percentages reflect the relative contribution of each subcellular fraction to all published studies. In total, 51% of all neuroproteomics studies that were included in this review make use of subcellular fractions.

The subtle changes in protein expression and their recruitment to specific compartments in dendritic spines require a reduction in sample complexity for specific changes in the synapse to be distinguished from background processes in the cell body. Several strategies exist for the fractionation of sample material to reduce complexity, and fractionation is used in more than 60% of all published proteomics studies focusing on the synapse. Synaptic fractions of synaptosomes, PSD, and the cytomatrix of the active zone can be obtained by Percoll or sucrose gradient centrifugation steps in well-described protocols (1). Although these protocols allow for in-depth characterization of these specific cellular components, information on intercompartmental interactions, such as filamentous actin remodeling in dendritic spines, is lost. Moreover, a recent study comparing different synaptosome preparation protocols revealed considerable variability in synaptosome purity and types of contamination (57). It is therefore recommended to thoroughly examine the most appropriate isolation technique for each proteomics study. A commonly used practice in the study of axonal proteomes is the use of compartmentalized chambers that separate axons from the cell body, allowing the study of the axonal proteome (58, 59, 60). Figure 2 illustrates the most commonly studied subcellular structures. Such studies are still limited because of the need for robust purification strategies, which are not available for the majority of subcellular structures.

#### Affinity Purification MS

Additional sample preparation steps are required when analyzing PPIs because classical proteomics workflows do not yield information on the interaction partners of the protein at the time of lysis. Furthermore, PPIs can be dynamic and transient and therefore call for the use of tailored enrichment methods. When antibodies are available, enrichment can be achieved in near-physiological conditions, making it a popular workflow for the study of protein complexes (61). Affinity purification–MS (AP-MS) experiments can be performed with immobilized antibodies, proteins, peptides, or ligands to isolate protein complexes.

Using antibodies, several postsynaptic complexes have been investigated, including several α-amino-3-hydroxy-5-methyl-4-isoxazolepropionic acid (AMPA) receptor subunits (62, 63), native AMPA receptor complexes (64), N-methyl-D-aspartate (NMDA) receptor subunits (62, 65), and post synaptic density 95 (66). Immunoprecipitation of mGluR1 and mGluR5 in mouse hippocampal and cortical lysates showed that in both regions, mGluR1/5 engages in direct interaction on the postsynapse (67). An overview of proteomics analyses of postsynaptic protein complexes in relation to neuronal plasticity can be found in (68, 69).

Although traditionally less well studied, several efforts have recently been made to elucidate inhibitory synapse complexes. Using AP-MS from transgenic mice with a tagged GABA_A_ receptor ɣ2 subunit, Ge *et al.* identified and characterized GABA_A_ receptor–associated proteins that are involved in regulation of surface expression and inhibitory homeostatic plasticity, such as cleft lip and palate transmembrane protein 1 (70, 71). Another recent AP-MS study in *Caenorhabditis* *elegans* identified the *O*-GlcNAc transferase OGT-1 to be an important cofactor in GABA neuron function (72).

For transient and weak interactions, however, detection is limited to classical approaches. Elegant solutions for these technical limitations are the proximity-based labeling approaches.

#### Proximity Labeling Approaches

**Fig. 3:**
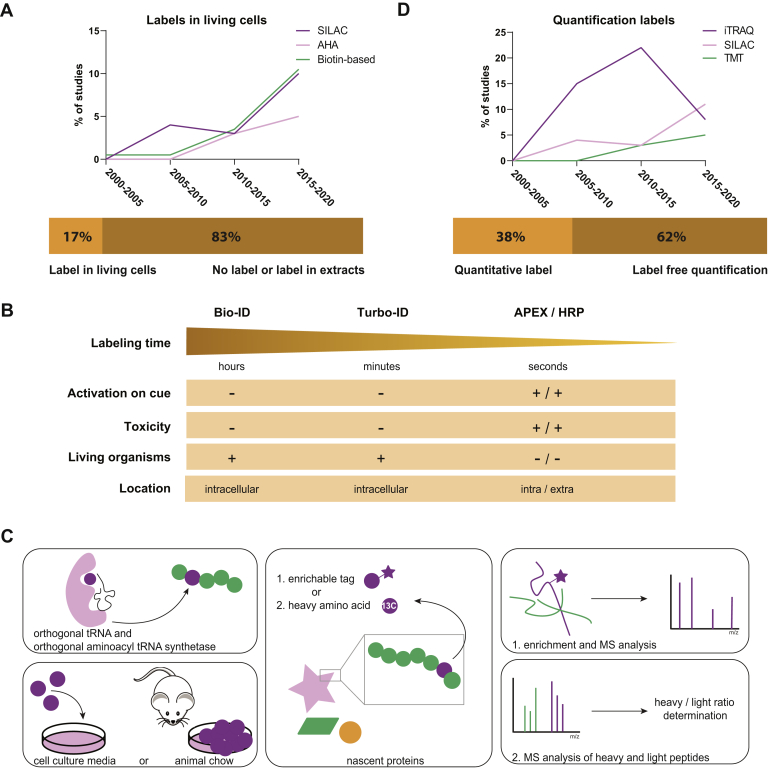
**Overview of bio-orthogonal and proximity labeling approaches and prevalence of labels in neuroproteomics studies.***A*, prevalence of the use of enrichment labels. In the last decade, an increase in labeling under native conditions (*i.e.*, in living cells or even in living organisms) for labeling of specific cellular structures and/or cellular processes such as protein translation can be observed. *B*, characteristics of the most prominently used proximity labeling constructs. *C*, workflow of bio-orthogonal labeling experiments. Unnatural amino acids can be genetically introduced using orthogonal tRNA and an orthogonal aminoacyl-tRNA synthetase (*upper panel*) or supplemented to cell culture media or animal chow (*lower panel*). The unnatural amino acid can contain an enrichable tag, or a heavy isotope, so that nascent proteins can be enriched and subsequently analyzed by MS or heavy isotope–containing proteins can be analyzed simultaneously with existing, natural proteins, after which the ratio between heavy and light proteins can be determined. *D*, prevalence of the use of quantification labels.

Several proximity-based labeling methods have been developed in recent years that can be used to map any (membrane-bound) microdomain of a cell. These methods are based on the fusion of a protein of interest to enzymes that can generate a reactive protein label, most commonly biotin, in living cells (73). These protein labels can subsequently be used for isolation of protein complexes or molecular ‘environments’ of the protein of interest, for instance, with streptavidin-coated beads, and their use in neuroproteomics studies is increasing (Fig. 3*A*).

The most commonly used proximity-dependent protein biotinylation methods are horseradish peroxidase (HRP), proximity-dependent biotin identification (BioID) and the more recently developed successor TurboID, and the engineered ascorbate peroxidase (APEX). Because the labeling radius of these enzymes is limited (around 10 nm), they can be very suitable to map the population of proteins within a specific structure, as well as their spatial distribution (73). In BioID, a promiscuous biotin ligase is fused to a distinct subcellular compartment by fusion with a strong targeting motif, which labels proximal proteins in a couple of hours after the addition of a high concentration of biotin (74). Using *in vivo* BioID, researchers have accomplished *in vivo* biotinylation of both excitatory and inhibitory synaptic protein complexes in the mouse brain (75). As biotinylation occurred in a native environment, many notoriously difficult proteins, such as membrane proteins, could be identified. TurboID is the result of the directed evolution of BioID’s BirA enzyme, decreasing labeling time to 10 min (76). Both peroxidase-based approaches, HRP and APEX, require incubation with a biotin aryl azide such as biotin phenol and subsequent labeling initiation by addition of H_2_O_2_. The peroxidase creates a phenoxyl radical with the possibility to covalently tag proximal proteins at electron-rich amino acid side chains, such as tyrosine (77, 78, 79). To be considered, however, is the toxicity of H_2_O_2_ for living cells. Because the reactive intermediate’s half-life in this reaction is much shorter than in BioID approaches, the biotinylation reaction is much faster, and a smaller, more accurate labeling radius is created. This increase in sensitivity allows making ‘snapshots’ of protein interactions over time so that protein dynamic interactions, as well as cellular localization of the protein of interest, can be deduced (80, 81). APEX labeling of α-synuclein in cortical neurons led to the identification of mRNA translation, endocytosis, and synaptic transmission proteins, indicating that alterations of these pathways in Parkinson’s disease could be directly related to α-synuclein spatial localization (82).

For the labeling of extracellular compartments such as synaptic clefts, HRP-fusion constructs, which cannot be used in many intracellular compartments because of their reducing environments, were designed. Using a cell membrane–impermeable biotin phenol conjugation, proteomes of both excitatory glutamatergic and inhibitory GABAergic synaptic clefts were created (80, 83). A split HRP was created to study intercellular PPIs, in which two inactive HRP-fusion constructs are activated upon colocalization of their fusion proteins. Fusion to presynaptically localized neurexin, in combination with postsynaptic neuroligin then allowed for synapse detection between two predefined sets of neurons, as demonstrated *in vivo* in mouse retinal ganglion cells (84). The labeling of transcellular protein interactions was enabled by the development of Split-TurboID, in which N- and C-terminal TurboID fragments were directed to the extracellular surface of neurons and astrocytes, respectively. When in close proximity, enzymatic activity is recovered and biotinylation of proximity proteins occurs. Interestingly, this approach was applied in the living mouse cortex, after local biotin injection (56).

Taken together, proximity labeling approaches have been embraced by the proteomics community as the new standard for the study of PPIs, as compared with antibody-based affinity approaches. Figure 3*B* contains an overview of the most commonly used proximity labeling constructs. However, several limitations have to be taken into consideration. As the construct needs to be inserted into cells, the bait protein is not the endogenous protein but the fused protein of interest and the enzyme. Transfection protocols need to be optimized to ensure both transfection efficiency and levels of protein of interest so that it is comparable to ‘native’ conditions. Moreover, during construct development, one has to keep in mind that the enzyme placement does not interfere with protein functionality and localization.

#### Crosslinking MS

For the study of protein complexes, chemical crosslinking MS (XL-MS) has emerged as a powerful addition to classical AP-MS experiments. In XL-MS experiments, two proximate amino acid residues, most often lysine, are covalently bound by a crosslinking molecule. This crosslinker typically consists of two functionally reactive groups, separated by a spacer. Crosslinked residues are identified *via* MS, and a distance constraint is determined. The distance constraint is determined *via* the sum of the length of the spacer arm and the side chains of the amino acid residues (85, 86). The resulting crosslink data contain information on both intraprotein and interprotein interactions, where crosslinked residues originated from the same or different proteins, respectively. XL-MS is therefore very suitable to reveal detailed information on PPIs and yields additional information on the structure of a protein and protein complexes. However, most crosslinking reagents are not cell permeable, limiting the use of XL-MS to cell lysates for the time being. Using XL-MS, an elaborate interaction atlas of more than 2000 proteins was made of the mouse synapse, using pooled microsome and synaptosomal fractions of hippocampal and cerebellar tissue. Next to extensive information on PPIs, the obtained datasets were used to elucidate specific PPI sites of SNARE proteins, to model the auxiliary AMPAR interaction complex and conformational changes of specific kinase domains (87).

Elucidation of protein complexes in the synapse at spatiotemporal resolution is essential in the journey toward single-synapse proteomic profiling, which was emphasized by the creation of the Mouse Lifespan Synaptome Atlas (88). Here, a tremendous effort was made to characterize single-synapse compositions of excitatory synapses in more than 100 brain regions in the developing mouse brain, resulting in a publicly available brain-wide atlas of synaptosomes. The atlas was created combing a semiautomated imaging platform of two fluorescently labeled constituents of multiprotein scaffolding complexes, post synaptic density 95 and SAP102, the localization of which led to the classification of 37 subtypes of excitatory synapses (88, 89).

### Protein Synthesis and Turnover Dynamics

#### Bio-Orthogonal Labeling Approaches

The study of dynamic protein expression was aided significantly by the introduction of bio-orthogonal labeling approaches, in which cell culture media are supplemented with unnatural amino acids. These can have heavy-labeled carbon and/or nitrogen, such as in Stable Isotope Labeling by Amino acids in Cell culture (SILAC), or are modified with an azide or alkyne moiety, such as azidohomoalanine (AHA). Incorporation of AHA in newly synthesized proteins then enables for click chemistry–based enrichment upon lysis, followed by identification and relative quantification of differential protein expression (Fig. 3*C*). The combination with (pulse) SILAC adds an additional layer of confidence on the observation of truly newly synthesized proteins. The use of such bio-orthogonal labeling approaches in living cells or organisms is increasing (Fig. 3*A*) and is especially interesting in synapse proteomics studies where perturbations in protein expression are thought to be small. Advantages of heavy isotope labeling include the possibility to pool multiple samples into one MS measurement, whereas click chemistry–based approaches enable enrichment of proteins of interest, which aids identification of low abundant proteins and allows for the use of smaller sample amounts. Potential hurdles in the use of these labeling approaches include low metabolic flux in terminally differentiated cells, generally leading to reduced labeling efficiency (90, 91). Moreover, depletion of amino acid storage by complete media change is not recommended because of the pivotal role of secreted factors such as neurotransmitters in neuronal health. For the use of short-pulse experiments, however, these can be overcome by a competitively high addition of the noncanonical amino acid in the preconditioned media. Moreover, mathematical models have been proposed to normalize for discrepancies in labeling efficiency (92). Because the spatial distribution of methionine does not allow for all tryptic peptides to contain an AHA upon metabolic labeling experiments, extra control conditions such as methionine controls are recommended for increased confidence. The combination pulse of SILAC and AHA allows for the enrichment of labeled proteins and relative quantification of enriched proteins *via* the heavy-labeled n-terminal arginine or lysine present on all tryptic peptides and is therefore often used. Although most studies have relied on label-free quantification approaches (62%), the use of SILAC and isobaric mass tags such as tandem mass tags has increased in popularity (Fig. 3*D*). This increase in popularity can mostly be attributed to the possibility of multiplexing of up to 16 samples in one MS measurement, thereby significantly decreasing analysis time. Another advantage of labeling lies in quantification, as the creation of a pooled reference sample that is spiked in each separate sample mix allows for correction of shifts in retention time and relative intensities. However, the addition of labeling reagents requires additional sample preparation steps, which increases sample variability. Moreover, the number of samples that can be multiplexed is still limited, which creates boundaries in the experimental design. Advancements in both the stability of analysis tools and data analysis software have increased the confidence in label-free quantification strategies.

A potential alternative that does not require depletion of culture media is O-propargyl-puromycin, an alkyne analog of puromycin. Puromycin, an aminonucleoside antibiotic and structural analog of an aminoacyl-tRNA, blocks protein synthesis *via* the formation of a nonhydrolyzable peptide bond in the elongating peptide. This terminates protein elongation and produces truncated, puromycin-modified peptides that with the use of O-propargyl-puromycin can be enriched and analyzed using MS. This approach, called SUnSET (93), was successfully applied *ex vivo* in axons to identify mechanistic target of rapamycin-initiated local protein synthesis after nerve injury (94). Although SUnSET was shown to not interfere with the protein synthesis rate (93), one has to keep in mind that the labeled products are in fact not functional proteins, but rather truncated peptides, which very likely affects cell homeostasis and cellular functioning.

In a recent study on the role of protein phosphorylation and translation during the induction and maintenance of mGluR5-induced LTD in hippocampal neurons, short pulses of AHA were used to identify more than 200 newly synthesized proteins (95). In another study, AHA was used to study protein synthesis during a 24-h synaptic scaling experiment. Hippocampal neurons were stimulated with either a Na^+^ channel antagonist for 24 h or a GABA_A_ receptor antagonist, to increase or decrease miniature excitatory postsynaptic current amplitude, respectively. Approximately 300 proteins were found to be differentially regulated, among which proteins involved in excitatory synapses and glutamate receptor complexes (96). A follow-up study using Bio-Orthogonal Non-Canonical Amino acid Tagging, where a temporal trajectory of the homeostatic scaling response was obtained showed that there was little overlap in newly synthesized proteins between an early (2 h) and late (24 h) time point, although similar general functional processes were regulated, indicating that slight alterations in the proteomic composition can affect the duration and polarization of synaptic remodeling (97). Moreover, stable isotope labeling and pulsed AHA were utilized in cultured glial cells derived from a mouse model of vanishing white matter to identify protein signaling and metabolic pathways affected by a common Eif2b mutation (98).

Incorporation of bio-orthogonal molecules is not limited to amino acids and could therefore also be used to study the dynamics of other biomolecules such as sugars and lipids. The addition of heavy-labeled or enzymatically modified sugars can be used to monitor glycosylation patterns in neurons (99), and heavy-labeled lipids and fatty acids are also readily available.

#### Protein degradation

Protein turnover is the net result of the synthesis of nascent proteins and the degradation of mature proteins. This equilibrium does not only allow for the replacement of damaged proteins but has been proven to be essential in dynamic cellular processes, such as synaptic plasticity (100). The role of ubiquitination and the ubiquitin–proteasome system in synaptic plasticity is reviewed in (101).

The combination of rapid protein turnover and the stability of long-lived synaptic proteins have been shown to be present in both presynaptic and postsynaptic compartments (reviewed in (102)). Using heavy-labeled lysine in mouse chow, followed by a 7-week chase with light-labeled lysine, it was recently shown that the majority of heavy-labeled proteins were rapidly degraded in the chase weeks. Cellular fractionation showed that protein turnover is higher in the cytosol than synaptosomes. Moreover, protein turnover was activity dependent, as determined in an enriched environment experiment, in which a group of mice underwent experience-dependent synaptic plasticity (103). Interestingly, in primary hippocampal cultures, neuronal protein turnover also seems to be influenced by the presence of extracellular matrix compartments, as well as other cell types such as glia. The same study also showed that presynaptic proteins tend to have longer half-lives than average and that that glutamate receptors exhibit shorter half-lives (104). Remarkably, inhibition of the proteasome by a variety of proteasome inhibitors does not affect degradation rates of the majority of synaptic proteins, as was determined in cortical neurons where a multiplexed SILAC approach was used to measure protein degradation. This seems to imply that many synaptic proteins are degraded *via* an alternative route. However, proteasome inhibition did seem to suppress the synthesis of synaptic proteins (105).

### PTMs and Protein Signaling

**Fig. 4:**
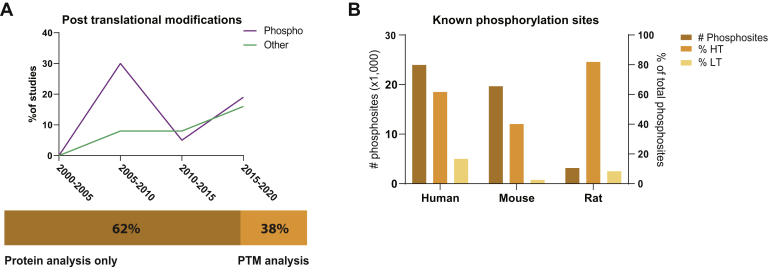
**Post-translational modifications in neuroproteomics studies focused on the synapse.***A*, only a minority of published studies have analyzed the prevalence of one or more PTMs in their proteomics dataset. Although phosphorylation is the most studied PTM, other PTMs such as N-glycosylation and ubiquitination are gaining interest. *B*, known phosphorylation sites and supporting evidence from high-throughput (HT) and low-throughput (LT) studies for the top three used model organisms in neuroproteomics studies. The data used in this graph were taken from the PhosphoSitePlus knowledgebase (133). PTMs, post-translational modifications.

The biological functionality of proteins is not solely dependent on their expression levels but can be regulated extensively *via* more than 100 different PTMs. Owing to the low stoichiometry of most PTMs, comprehensive analysis requires enrichment steps before mass spectrometric analysis. In the case of phosphorylation, this is often performed using immobilized metal affinity chromatography approaches in which peptides with a negatively charged phospho group are bound to iron. Although these strategies typically required milligrams of protein input material, and therefore hampered the analysis of phosphorylation dynamics in precious neuronal samples, recent advances have made it possible to perform sensitive and reproducible enrichment of phosphopeptides with less than 10 μg of protein input material (106). Adaptations to this protocol have led to the development of a strategy for the identification of mannose-6-phosphate–modified hydrolases. This low-abundant PTM is critical for the transport of newly synthesized hydrolases from the Golgi apparatus to the lysosome, where they exert their function. Together with selective triggering of the newly identified phosphomannose oxonium fragment marker ions, hundreds of mannose-6-phosphate–modified glycopeptides could be identified (107). Although phosphorylation is the most widely studied PTM, other PTMs have gained interest in the last decade (Fig. 4*A*).

#### Phosphorylation

The first studies on the synaptic phosphoproteome were performed on isolated mouse PSD (108, 109, 110), as well as human synaptosomal fractions (111), and identified approximately 300 phosphorylation sites on key synaptic proteins. With the advancements in technology, the number of detected phosphoproteins also increased, enabling the study of activity-dependent phosphorylation changes, such as the phosphorylation dynamic changes upon naïve and stimulated synaptosomal preparations (112, 113), as well as brain region–specific phosphorylation changes (114), and the discovery of a sequence-specific S-Q phosphorylation motif that is regulated during synaptic plasticity (115). Interestingly, this S-Q phosphoproteome was heavily dependent on GABA_A_ and NMDA receptor activity, whereas stimulation of metabotropic glutamate receptors (mGluRs) with (*S*)-3,5-Dihydroxyphenylglycine did not influence S-Q phosphorylation. Another recent study performed on primary hippocampal neurons analyzed the phosphorylation dynamics of mGluR-LTD over multiple time points and quantified over 5000 phosphorylation sites, mapping important kinases in synaptic plasticity and identifying new phosphoproteins involved in AMPA receptor trafficking in mGluR-induced LTD (95). A phosphoproteomics study on neuronal differentiation of SH-SY5Y neuroblastoma cells elegantly showed that upon neuronal differentiation cues, phosphorylation motifs of prominent cell-cycle division kinases were downregulated, whereas the relative contribution of G protein–coupled receptor kinases and calcium/calmodulin-dependent protein kinase 2 increased significantly (116). In the biggest neuronal phosphoproteomics study to date, the (phospho)proteomic effects of *in vivo* administration of several agonists and antagonists of the kappa opioid receptor were studied in four murine brain regions, resulting in the identification and quantification of an astonishing 50,000 phosphorylation sites (117). The acquired data resulted in the identification of time-dependent, as well as brain region–specific and stimulus-specific, phosphorylation patterns. Another study showed that 30% of the mouse synaptic phosphoproteome showed oscillatory patterns, indicating that phosphorylation of essential synaptic proteins, such as receptors and channels, and especially kinases, is key to essential circadian brain processes such as synaptic excitation and inhibition (118).

#### Other PTMs

A recent study by Smith *et al.* investigated the extent of S-nitrosylation (SNO) in rat cortical neuron nuclear extracts. SNO involves the attachment of a nitric oxide group to cysteine thiol residues. Not only did they identify more than 600 S-nitrosylated proteins but also they were able to generate several lysine-specific SNO motifs and found that SNO modification of the histone-binding protein RBBP7 was necessary for dendritogenesis (119). Palmitoylation (or S-acylation), the attachment of the 16-carbon saturated fatty acid palmitate to cysteines, was found to be crucial in neuronal functioning and trafficking of neuronal proteins (120, 121). From all newly identified palmitoylated proteins, many scaffolding and receptor proteins were identified, including NMDA receptor subunits. This is especially interesting because palmitoylation is, like phosphorylation, reversible and can therefore play an important role in receptor trafficking (120). As discussed previously, protein degradation plays a pivotal role in synapse biology and is therefore particularly interesting to study. Ubiquitination is a notoriously tricky PTM to enrich for MS analysis because the di-glycine motif that is typically used for enrichment is not specific and needs to be performed on peptide level, after digestion (110). Nevertheless, antibody-based enrichment of this motif has led to a considerable amount of key synaptic proteins (122), and significant differences in ubiquitination were observed in Huntington mouse brain samples (123). In an alternative approach, the BirA enzyme was fused to multiple copies of ubiquitin modified with a short N-terminal sequence that can be biotinylated. Next, the polyubiquitin is processed into individual ubiquitin molecules by endogenous deubiquitinating enzymes, which then allows this modified ubiquitin to be readily available for target proteins that can now also be biotinylated by BirA. Using this approach, ubiquitinated proteins in *Drosophila* embryonic and adult neurons were compared, as well as specific targets E3 ligases Parkin and Ube3a (124, 125).

Glycosylation is one of the most common and, at the same time, one of the most complex PTMs. Classical challenges in the study of PTMs, such as the need for enrichment, do not apply here. In fact, the high abundance and heterogeneity of the modification are the major hurdle in accurate analysis (126, 127). Recent advancements, such as the extension of the mass range during electron transfer higher-energy collisional dissociation (128) and the use of alternative dissociation strategies such as activated-ion electron transfer dissociation (129) have led to a significant increase in N-glycopeptide identification and N-glycosite profile mapping, respectively. Using a cerebellum-specific KO mouse for *Srd5a3*, a gene that is involved in the initiation steps of N-glycosylation, Medina-Cano *et al.* discovered that especially highly glycosylated proteins were affected by the mutation, linking high N-glycan multiplicity to neurite outgrowth and axon guidance processes (127).

#### PTM Interplay

Interestingly, an emerging line of research is focused on the crosstalk, or interplay, of multiple PTMs on a single amino acid residue. Most prominently, a study on the crosstalk between O-GlcNAcylation and phosphorylation on murine synaptosomes found that more than 5% of their identified O-GlcNAcylated serine and threonine residues were also phosphorylated, and protein kinases were prominently enriched, indicating that the crosstalk could be regulating enzymatic activity (130). A challenge in the study of PTM crosstalk, however, is the necessity of enrichment before mass spectrometric analysis, limiting the possibility of detecting both PTMs in a single analysis.

### Data Analysis and Interpretation

Databases containing localization of new PTM sites has expanded tremendously with the arrival of large shotgun MS experiments and other high-throughput analysis methods. With the increase in detectable phosphorylation events comes the laborious task of data interpretation and validation. Only 5.3% of all identified human phosphorylation sites reported in the PhosphoSitePlus database that have been detected in high-throughput studies have a reported function, as determined by low-throughput validation studies (131). Low-throughput studies are needed to study the role of these prominently identified phosphorylation sites and to increase the validity of phosphoproteomics analyses. Studies of phosphorylation status increase in complexity through the fact that an increase in phosphorylation does not necessarily mean an increase in activity and vice versa. Even more complicated are phosphorylated proteins or even single peptides with multiple phosphorylation sites.

Because functional PTMs are likely to be evolutionarily conserved between species, it is an often-used criterion for selecting a specific phosphorylation event for further characterization. However, a comparison of PTMs between species can be complicated because many modification sites are located in disordered regions (132). Moreover, the rate of identification and functional characterization of phosphorylation sites is not linear across species, as illustrated in Figure 4*B*. In humans, almost 240,000 phosphosites have a reported function in PhosphoSitePlus, while they only represent 62% and 17% of the total identified phosphosites in high-throughput (HT) and low-throughput (LT) studies, respectively (133). Almost 80% fewer phosphosites are reported for rats, the model system most often used in neuroproteomics studies. This makes phosphoproteomics data interpretation and analysis significantly less informative and more laborious because efforts have to be made to translate gene, protein, and phosphorylation site data from humans to rats.

The study of multiple PTMs is hampered by the limitations in computational analysis. The immense amount of data that results from an MS-based proteomics experiment necessitates automated ways of data analysis and interpretation that goes beyond the annotation of spectra and database searching. Several data repositories have been created, specifically focused on the synaptic proteome, to structure the increasing amount of data (134, 135, 136). A frequently used strategy during the data interpretation process is the use of gene ontology enrichment analyses to gain insight into the overrepresentation of genes or proteins in the dataset involved in a particular biological process, with a specific molecular function, or present in a defined cellular component. Although its use has been helpful in the understanding of several big synaptic datasets (137), the lack of annotation and expert curation of synapse-specific gene and therefore gene products such as proteins limited its potential and interpretation of results. With the release of SynGO, ‘an interactive knowledge base that accumulates available research about synapse biology using gene ontology annotations to novel ontology terms’ (138), more than 1000 genes with localization or function in the synapse were annotated and expert-curated, improving the interpretation of large synaptic–OMICS datasets.

Moreover, analysis tools for integration and interpretation of phosphoproteomics data are being developed to deal with some of these challenges. PhosphOrtholog, for instance, was developed to map protein modification sites between species (132), and several tools have been developed to aid integration of kinase activation state with known targets and known PPIs (such as INKA (139) and PHOTON (140)), and visualization of dynamics in temporal phosphorylation datasets, such as the Cytoscape plugin PhosphoPath (141).

#### Computational Modeling

Genome-wide association studies have contributed tremendously to the identification of risk genes for many neurological disorders, including psychiatric disorders such as schizophrenia. However, in the majority of cases, these findings did not translate linearly with alterations in the proteome or neuronal phenotypes. Strategies combining several research techniques might contribute to our understanding of these complex, multifactorial diseases. Although the combination of RNA sequencing and proteomics is most often used, it is becoming increasingly clear that these are difficult to integrate and interpret. However, integration of mRNA and proteome datasets can contribute to our understanding of essential brain processes, as was shown in a study where both transcriptome and proteome alterations were followed during normal sleep and high sleep pressure in mice. Both the proteome and transcriptome showed circadian oscillations, but these almost completely abolished in the synaptic proteome during sleep deprivation, whereas the transcriptome was much less affected (142). A recently published study by Rosato *et al.* used a different strategy, combining so-called cellomics and proteomics experimental data to investigate neuronal phenotypes of schizophrenia risk genes. To this end, they studied the phenotypic alterations in primary cultured neurons upon knockdown of more than 40 candidate schizophrenia risk genes. They grouped the knockdown-induced phenotypes and performed proteomics analysis to identify the molecular pathway underlying the shared risk of these genes, thereby enhancing the understanding of the molecular fingerprint of schizophrenia (143). Approaches like these can greatly contribute to our knowledge on so-called synaptopathies, a term applied to diseases with synaptic dysregulation (reviewed in (144, 145)).

With the exponential increase in proteomics data from a variety of cell types, model systems, modifications, and perturbations that has been generated over the last decades, another challenge has emerged: the integration of these data not only to strengthen the knowledge that has already been gained but also to predict understanding of brain processes and circuitry complexity. Systems biology approaches, where biological data are combined with computational modeling and mathematics, are being developed. In short, more and more layers of complexity are added to the understanding of molecular networks that underlie brain function in both health and disease, as more and more information becomes available. For instance, the first layer exists of the classical neuronal signaling model, only considering the presynaptic and postsynaptic terminals. A second layer is created where information from glia cells is included, a third with molecular elements of the extracellular matrix, and then the neurovascular unit, the immune system, and so forth. Moreover, on a molecular level, information on expression, interactions, structural organization, and turnover of proteins creates additional layers of information that need to be taken into account. Modular systems biology tries to organize already available data of big datasets, including genomics, epigenomics, transcriptomics, proteomics, metabolomics, and others, into mathematical and computational models to get a more in-depth view of the mechanisms of complex biological processes. These include, but are not limited to, the identification of key pathways and even predictions of how different modules in a network will respond to perturbations in a system, such as synaptic plasticity. The most recent evidence of the major pathways that should be considered in the development of a modular and computational model of synapse formation and functioning is reviewed in (54).

### New Techniques

The wide variety of cell types in the brain poses one of the major challenges in the neuroproteomics field. The majority of experimental studies focusing on synapse biology require the use of either brain tissue samples or cultured primary neurons, which both contain a plethora of different cell types. The possibility of selective enrichment of specifically defined cell populations could significantly aid the neuroproteomics field. A big step forward was made with the introduction of inducible genetic labels. Using a Cre-recombinase system to express a methionyl-tRNA synthetase with an expanded amino acid binding site, researchers are now able to label specific cell types using cell type–specific promotors. Methionyl-tRNA synthetase enables the methionine tRNA to be charged with the unnatural azidonorleucine, which can then be easily enriched using classic click-chemistry methods (146). In neurons, a similar approach using *trans*-cyclooct-2-ene (TCO∗)-modified L-lysine (TCO∗-A) was introduced into different transmembrane AMPAR regulatory proteins to study transmembrane AMPAR regulatory protein modulation over AMPA receptors in living neurons using fluorescence microscopy (147). Similarly, cell type–specific expression of proximity labeling constructs can achieve cell type–specific labeling of PPIs (148). It has to be noted, however, that these approaches can only be applied successfully if one can find a cell type–specific protein to label.

#### Single-Cell Proteomics

Processes such as long-term potentiation or LTD are known to be cell and even synapse specific, and the possibility of measuring at single-cell sensitivity could therefore contribute to our understanding of synaptic plasticity on a molecular level. Next to the obvious benefits (less sample material is needed, less variation is expected because of increased homogeneity of the obtained sample material), confidence in the obtained results is expected to increase because many more biological replicates can be measured. One of the biggest challenges in single-cell proteomics is the detection of proteins that are present in low copy numbers. In addition to instrumental improvements, such as a faster duty cycle, research focuses on decreasing sample complexity by fractionation more extensively before MS analysis. Traditionally, an orthogonal separation is used offline, before standard online reversed-phase LC-MS/MS, such as high pH or size-exclusion chromatography. Alternatively, Choi *et al.* have recently developed a near single-cell method with trace-level sensitivity by coupling offline reversed-phase fractionation and capillary electrophoresis MS. This approach allowed for the detection of more than 700 protein groups using only 1 ng of protein digest, the equivalent of five neurons (149).

#### Targeted MS

Concerning MS analysis, we can see some general trends in the direction of parallel reaction monitoring (PRM) and selected reaction monitoring (SRM) types of analysis. Advantages of these targeted assays include increased sensitivity and accuracy as compared with traditional discovery-based methods, where the stochastic selection of ions often leads to incomplete information and a bias toward a subset of proteins (150). SRM was successfully used to study a subset of synaptic proteins in the cerebrospinal fluid of Alzheimer’s disease patient cohorts, where synaptic proteins were found to be reduced already in preclinical Alzheimer’s disease, preceding clinical symptoms (151). Alternatively, quantification of synaptic proteins of interest can be improved using PRM, as was shown in PSD fractions of WT *versus* Shank3B cortical tissue (152). Compared with SRM, PRM is based on the isolation of a preset precursor ion, after which fragments, or transitions, are measured and used for quantification. However, instead of a triple quadrupole setup, an orbitrap replaces the third quadrupole, and unlike SRM, all transitions of a given precursor ion are scanned, that is, parallel monitoring of all fragments takes place (153). Because PRM assays can be performed on the more commonly used Q Exactive mass spectrometers, it is a promising addition to the neuroproteomics toolbox.

In summary, 2 decades of synapse proteomics research, with the identification of more than 2000 synapse proteins, tens of thousands of phosphorylation sites, transient and time-resolved information on PPIs and structures, has significantly increased our knowledge of the molecular composition and functioning of the synapse. Moreover, with the majority of MS-based proteomics datasets freely available in data repositories such as ProteomeXchange (154), the neuroscience community has gained a variety of valuable data resources. To advance the field of neuroproteomics further, the combination of spatial and temporal information is essential. Most improvements can be achieved in the experimental steps preceding typical proteomics sample preparation and begin with the choice of the organism, brain region, and sample type. Increased sensitivity of instrumentation has significantly decreased the amount of sample material needed for proteomics analysis. This now allows for the use of single-cell types, such as differentiated iPSCs and primary neuronal cultures, as well as for the enrichment of low abundant proteins and subcellular structures. In the last decade, there has been constant improvement in enrichment methods, both in living organisms or cells (such as proximity labeling approaches and noncanonical amino acid) and sample preparation processes (such as the enrichment of PTMs). Indeed, we can see a clear shift in the use of both of these areas (Table 1), thereby constantly increasing our knowledge of activity- and compartment-dependent protein expression, modification, and interaction profiles in synaptic compartments, and even in cell type–specific synapses.

**Table 1:** Overview of neuroproteomics studies using labeling techniques in living systems

Labeling technique	Reference	Organism	Sample type	Brain region	Cellular compartment
BioID	(75)	Mouse	Tissue	Hippocampus cortex	N.A.
	(124)	Fly	Homogenates	N.A.	N.A.
HRP	(83)	Rat	Primary neurons	Cortex	N.A.
	(80)	Rat	Primary neurons	Cortex	N.A.
APEX	(82)	Rat	Primary neurons	Cortex	N.A.
TurboID	(56)	Mouse	Tissue	Cortex	N.A.
SILAC	(104)	Rat	Primary neurons and glia	Hippocampus	N.A.
	(59)	*Xenopus laevis*	Cultured eyes	N.A.	Axon
	(58)	*Xenopus laevis*	Cultured eyes	N.A.	Axon
	(155)	Rat	Primary neurons	Hippocampus Cortex	N.A.
	(105)	Rat	Primary neurons	Hippocampus	N.A.
AHA	(96)	Rat	Primary neurons	Hippocampus	N.A.
	(97)	Rat	Primary neurons	Hippocampus	N.A.
	(95)	Rat	Primary neurons	Hippocampus	N.A.
15N	(156)	Rat	Tissue	Whole brain	Nucleus, endoplasmic reticulum, cytoplasm, mitochondria
	(157)	Mouse	Tissue	Barrel cortex	Synaptosome
ANL	(146)	Mouse	Tissue or extracts	N.S.	N.S.
FASS	(31)	Mouse	Tissue	Forebrain	Synaptosome

ANL, azidonorleucine; FASS, fluorescence-activated synaptosome sorting; N.A., not applicable; N.S., not specified.

## Conflict of interest

The authors declare no competing interests.
